# A Comprehensive Study on Pyrolysis Mechanism of Substituted β-*O*-4 Type Lignin Dimers

**DOI:** 10.3390/ijms18112364

**Published:** 2017-11-09

**Authors:** Xiaoyan Jiang, Qiang Lu, Bin Hu, Ji Liu, Changqing Dong, Yongping Yang

**Affiliations:** National Engineering Laboratory for Biomass Power Generation Equipment, North China Electric Power University, Beijing 102206, China; jiangxiaoyan@ncepu.edu.cn (X.J.); hubin@ncepu.edu.cn (B.H.); liujipower@ncepu.edu.cn (J.L.); dongcq@ncepu.edu.cn (C.D.); yyp@ncepu.edu.cn (Y.Y.)

**Keywords:** lignin model compound, β-*O*-4 linkage, functional group, pyrolysis mechanism, density functional theory

## Abstract

In order to understand the pyrolysis mechanism of β-*O*-4 type lignin dimers, a pyrolysis model is proposed which considers the effects of functional groups (hydroxyl, hydroxymethyl and methoxyl) on the alkyl side chain and aromatic ring. Furthermore, five specific β-*O*-4 type lignin dimer model compounds are selected to investigate their integrated pyrolysis mechanism by density functional theory (DFT) methods, to further understand and verify the proposed pyrolysis model. The results indicate that a total of 11 pyrolysis mechanisms, including both concerted mechanisms and homolytic mechanisms, might occur for the initial pyrolysis of the β-*O*-4 type lignin dimers. Concerted mechanisms are predominant as compared with homolytic mechanisms throughout unimolecular decomposition pathways. The competitiveness of the eleven pyrolysis mechanisms are revealed via different model compounds, and the proposed pyrolysis model is ranked in full consideration of functional groups effects. The proposed pyrolysis model can provide a theoretical basis to predict the reaction pathways and products during the pyrolysis process of β-*O*-4 type lignin dimers.

## 1. Introduction

Lignin is the most abundant renewable aromatic biopolymer in nature, biosynthesized through polymerization of three basic monolignols, i.e., *p*-coumaryl, coniferyl, and sinapyl alcohols which are interlinked by C–O bonds (e.g*.*, β-*O*-4, α-*O*-4 and 4-*O*-5) and C–C bonds (e.g., β-1, β-5 and 5-5) [1,2,3,4]. Apart from different linkages, there are various functional groups on the alkyl side chain and aromatic ring of the lignin basic units, including methoxyl, hydroxyl, hydroxymethyl, carbonyl and more [5,6,7]. Among them, methoxyl, hydroxyl and hydroxymethyl groups are native substituents while others (such as carbonyls) are non-native and chemically modified via the oxidization of the OH group on the C_α_ or C_γ_ positions [1,6]. Therefore, lignin is the most complex component among the three basic components of lignocellulosic biomass. Pyrolysis is a promising thermo-chemical conversion method for the utilization of lignocellulosic biomass materials [8,9,10], through which lignin can be decomposed to form various value-added aromatic compounds [9,11,12]. However, conventional pyrolysis is a non-selective process, and it has difficulty selectively controlling the lignin decomposition process towards specific aromatic compounds. In order to develop more efficient selective pyrolysis techniques, lignin pyrolysis mechanism and products formation pathways should be deeply uncovered to help provide a theoretical basis for directional control of the lignin pyrolysis process. Density functional theory (DFT) method is an effective tool to reveal the lignin pyrolysis mechanism at a micro level [13,14,15,16].

Due to the complex structure of natural lignin, lignin-based model compounds are widely used to investigate the pyrolysis mechanism of lignin. Considering that the β-*O*-4 linkage is dominant, accounting for about half of the linkages in lignin, β-*O*-4 type lignin dimer model compounds are typically selected for theoretical studies [13,17,18,19]. Previous research has investigated the pyrolysis mechanism of various β-*O*-4 type lignin dimer model compounds containing different functional groups on the alkyl side chain and aromatic ring [20,21]. Beste et al. [20] considered that phenethyl phenyl ether (PPE) mainly underwent C_β_–O bond and C_α_–C_β_ bond homolytic cleavage reactions during the primary pyrolysis process. However, Elder et al. [21] found that two concerted reactions (retro-ene fragmentation and Maccoll elimination) were prior to C_β_–O bond and C_α_–C_β_ bond homolytic cleavage reactions, and the energy barrier of retro-ene fragmentation was lower than that of Maccoll elimination. Currently, it is widely recognized that concerted reactions and homolytic reactions are coexisting during the lignin pyrolysis process [19,22]. Nevertheless, functional groups on the alkyl side chain and aromatic ring may have great effects on lignin pyrolysis mechanism. Basically, they can influence the bond dissociation energies (BDEs) of major bonds [5,6,17] and enrich the reaction pathways, including both concerted reaction pathways and homolytic reaction pathways [13,23]. Some experimental studies have also confirmed that functional groups could affect the thermal behavior and product distribution of lignin [24,25,26,27]. Kawamoto et al. [24,27] studied the effect of hydroxyl groups at C_α_ and C_γ_ positions of the alkyl side chain on pyrolytic reactions of β-*O*-4 type lignin dimer model compounds. Results suggested that the hydrogen bond between the two hydroxyl groups would affect the reaction sequences of C_γ_–elimination and C_β_–O bond homolytic cleavage, and further influence the product distribution. Britt et al. [28] found that the methoxyl group on the aromatic ring would weaken the C_β_–O bond and make it easier to undergo homolytic reaction. Furthermore, the methoxyl group would promote O–CH_3_ bond homolytic cleavage [23,29], and the hydroxyl group on the alkyl side chain would transform into ketone or accelerate the dehydration reaction [13]. However, there are few comprehensive studies focusing on the effects of these substituents on the unimolecular decomposition pathways of lignin.

Due to the limitation of previous studies, a pyrolysis model for β-*O*-4 type lignin dimers is proposed which takes the effects of native substituents (hydroxyl, hydroxymethyl and methoxyl) located on alkyl side chain and aromatic ring into consideration. The above three substituents are very common and characteristic in the natural lignin structure [6,17]. Furthermore, five specific β-*O*-4 type lignin dimer model compounds containing hydroxyl, hydroxymethyl and methoxyl groups are selected to reveal their pyrolysis mechanism by DFT calculation and to understand the proposed pyrolysis model.

## 2. Results

### 2.1. Pyrolysis Model for β-O-4 Type Lignin Dimers

**Figure 1:**
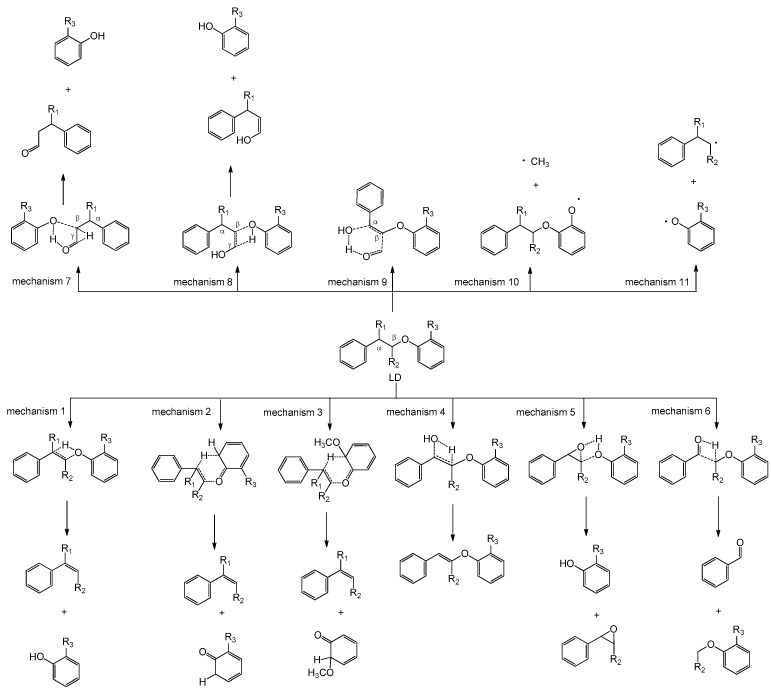
Pyrolysis model for β-*O*-4 type lignin dimers (LD).

Based on the possible pyrolysis reactions and previous studies [13,23,29], a pyrolysis model for β-*O*-4 type lignin dimers (LD) is proposed as shown in Figure 1, which considers the effects of hydroxyl, hydroxymethyl, and methoxyl groups on pyrolysis pathways. Mechanisms 1–9 are concerted reactions, while mechanisms 10 and 11 are homolytic reactions. Among them, mechanisms 1, 2 and 11 are three basic pyrolysis mechanisms for all β-*O*-4 type lignin dimers regardless of substituents. They are Maccoll elimination, retro-ene fragmentation and C_β_–O bond homolytic cleavage, respectively [21].

When the methoxyl group is located on the aromatic ring of β-*O*-4 type lignin dimer model compounds, mechanisms 3 and 10 will take place. Mechanism 3 is another retro-ene fragmentation. The H atom at C_α_ position transfers to aromatic carbon atom where the methoxyl group is located. Mechanism 10 is O–CH_3_ bond homolytic cleavage reaction. Britt et al. [29] found that O–CH_3_ bond as well as C_β_–O bond were the weakest bonds in lignin which were easy to break during the primary pyrolysis process.

When the hydroxyl group substitutes at C_α_ position, mechanisms 4, 5 and 6 will occur. Mechanism 4 is a dehydration reaction. The hydroxyl group at C_α_ position and the H atom at C_β_ position undergo dehydration reaction to form a compound containing an unsaturated C=C double bond. In mechanisms 5 and 6, the H atom of the hydroxyl group at C_α_ position will transfer to the oxygen atom of ether bond and C_β_ position meanwhile rupturing the C_β_–O and C_α_–C_β_ bonds, respectively.

When the hydroxymethyl group substitutes at C_β_ position, mechanisms 7 and 8 will take place through which the H atoms of hydroxyl group and C_γ_ position will transfer to the oxygen atom of ether bond with the rupture of C_β_–O bond, respectively. When the hydroxyl and hydroxymethyl groups are located on the C_α_ and C_β_ positions of a β-*O*-4 type lignin dimer model compound, it will undergo mechanism 9 to form water, formaldehyde and a compound with an unsaturated C=C double bond.

The pyrolysis model in Figure 1 depicts possible pyrolysis pathways of β-*O*-4 type lignin dimers during the primary pyrolysis process, which just provides the basic information to predict possible pyrolytic products formed in the subsequent pyrolysis process. In the following section, five specific model compounds will be employed to investigate their detailed pyrolysis pathways based on this model, to illustrate the competitiveness of possible pyrolytic pathways.

### 2.2. Pyrolysis Mechanism of β-O-4 Type Lignin Dimers Based on the Pyrolysis Model

In order to further understand the proposed pyrolysis model and explore the effects of hydroxyl, hydroxymethyl and methoxyl groups on the unimolecular decomposition pathways of β-*O*-4 type lignin dimers, five specific model compounds are selected to investigate their detailed pyrolysis mechanisms and pathways, including phenethyl phenyl ether (PPE), 1-methoxy-2-phenethoxybenzene (*o*-CH_3_O-PPE), 2-phenoxy-1-phenylethanol (α-OH-PPE), 2-phenoxy-3-phenylpropan-1-ol (β-CH_2_OH-PPE), and 2-(2-methoxyphenoxy)-1-phenylpropane-1,3-diol (α-OH-β-CH_2_OH-*o*-CH_3_O-PPE). The detailed integrated pyrolysis mechanism of α-OH-β-CH_2_OH-*o*-CH_3_O-PPE is discussed below and categorized into concerted mechanisms and homolytic mechanisms. Others are shown in the Supplementary Materials (Figures S1–S4).

#### 2.2.1. Concerted Mechanisms of Model Compound α-OH-β-CH_2_OH-*o*-CH_3_O-PPE

**Figure 2:**
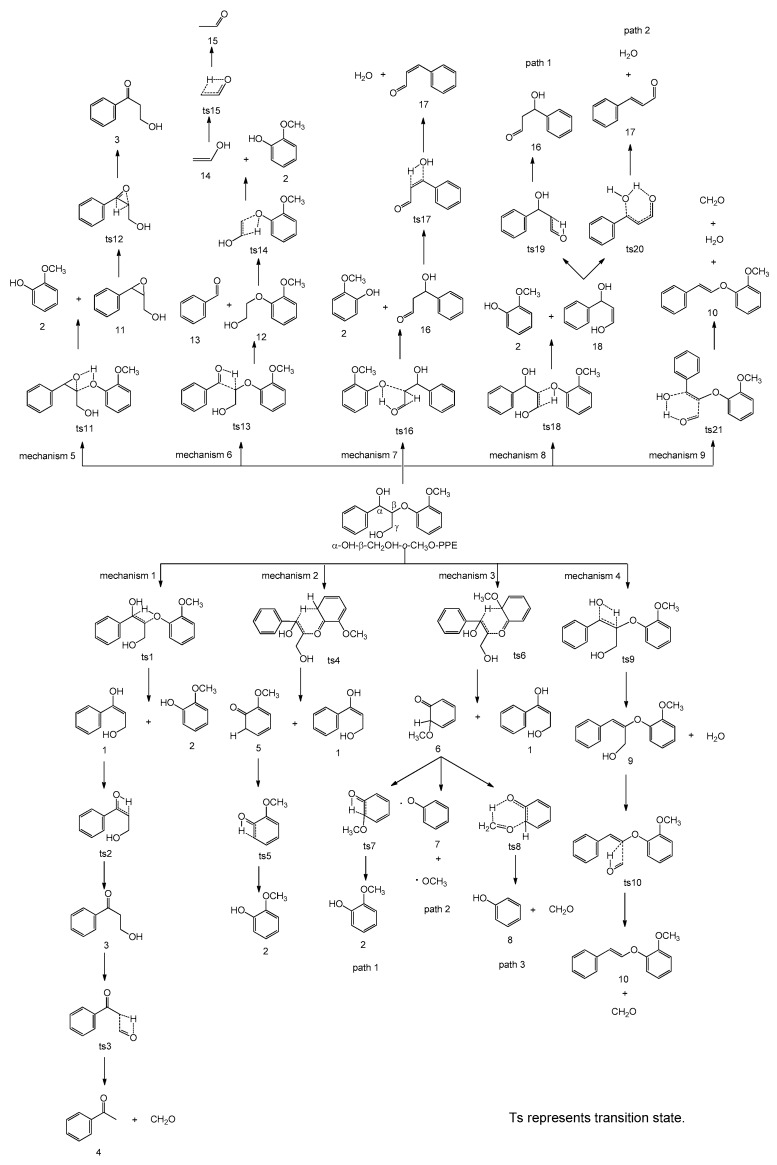
Possible reaction pathways based on concerted mechanisms 1–9 of the β-*O*-4 type lignin dimer model compound α-OH-β-CH_2_OH-*o*-CH_3_O-PPE.

**Figure 3:**
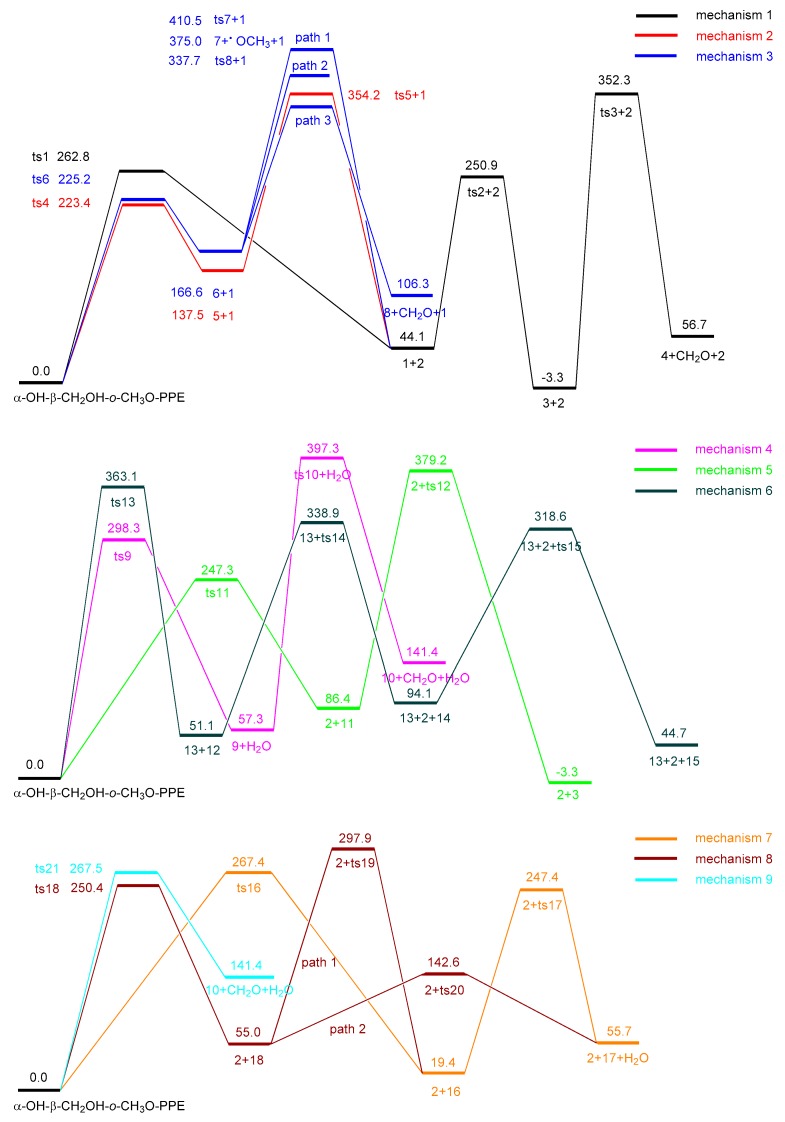
Potential energy profile along the reaction pathways based on concerted mechanisms 1–9.

Pyrolysis of model compound α-OH-β-CH_2_OH-*o*-CH_3_O-PPE involves nine possible concerted mechanisms (mechanisms 1–9 in Figure 1) due to the presence of the substituents. The possible reaction pathways following the nine concerted mechanisms are designed in Figure 2 and the potential energy profile along the pathways is shown in Figure 3.

Mechanisms 1 and 2 are the two common concerted mechanisms existing in the pyrolysis process of all β-*O*-4 type lignin dimers [21]. In concerted mechanism 1, α-OH-β-CH_2_OH-*o*-CH_3_O-PPE undergoes Maccoll elimination through a four-membered ring transition state **ts1** with an energy barrier of 262.8 kJ/mol. The H atom at C_α_ position transfers to the oxygen atom of the ether bond. Simultaneously, the C_β_–O bond ruptures and the C_α_–C_β_ bond transforms into C=C double bond, forming intermediate **1** and product **2** (2-methoxyphenol). Intermediate **1** contains an extremely unstable enol structure which easily undergoes isomerization through transition state **ts2** to generate product **3** (3-hydroxy-1-phenylpropan-1-one) with a ketone structure, overcoming an energy barrier of 206.8 kJ/mol. Product **3** with the hydroxymethyl group at C_β_ position may undergo a secondary reaction via a four-membered ring transition state **ts3** to form product **4** (acetophenone) and formaldehyde, overcoming an energy barrier of 355.6 kJ/mol.

In concerted mechanism 2, α-OH-β-CH_2_OH-*o*-CH_3_O-PPE firstly undergoes retro-ene fragmentation through a six-membered ring transition state **ts4** to form intermediates **1** and **5** with an energy barrier of 223.4 kJ/mol, which is lower than the energy barrier of **ts1** in concerted mechanism 1. Intermediate **5** then undergoes intramolecular hydrogen transfer reaction through transition state **ts5** to form product **2** (2-methoxyphenol) with an energy barrier of 216.7 kJ/mol. Intermediate **1** will transform into product **3** (3-hydroxy-1-phenylpropan-1-one) following the reaction pathway in concerted mechanism 1.

Concerted mechanism 3 is similar to concerted mechanism 2. The only difference is that the H atom at C_α_ position transfers to the aromatic carbon atom where methoxyl group is located through transition state **ts6** with an energy barrier of 225.2 kJ/mol, forming intermediates **1** and **6**. The energy barrier of **ts6** is close to that of **ts4** in concerted mechanism 2. Intermediate **6** has three possible subsequent reaction pathways. In path 1, it undergoes intramolecular hydrogen transfer reaction through transition state **ts7** to form product **2** (2-methoxyphenol) with an energy barrier of 243.9 kJ/mol. Due to the existence of the methoxyl group, intermediate **6** can also undergo paths 2 and 3. In path 2, it undergoes demethoxylation to generate radical **7** with an energy barrier of 208.4 kJ/mol. In path 3, it undergoes intramolecular hydrogen transfer reaction through a six-membered ring transition state **ts8** to form product **8** (phenol) and formaldehyde, overcoming the lowest energy barrier of 171.1 kJ/mol. Therefore, path 3 is most likely to occur among the three possible subsequent reaction pathways in concerted mechanism 3.

Mechanisms 4–6 are closely related to the hydroxyl group at C_α_ position. In concerted mechanism 4, the hydroxyl group at C_α_ position and the H atom at C_β_ position of α-OH-β-CH_2_OH-*o*-CH_3_O-PPE undergo dehydration reaction through a four-membered ring transition state **ts9** to form intermediate **9**, overcoming an energy barrier of 298.3 kJ/mol. Then intermediate **9** will lose a molecule of formaldehyde through transition state **ts10** to form product **10** (1-methoxy-2-(styryloxy)benzene) with an energy barrier of 340.0 kJ/mol due to the hydroxymethyl group at C_β_ position.

In concerted mechanism 5, α-OH-β-CH_2_OH-*o*-CH_3_O-PPE goes through transition state **ts11** to form product **2** (2-methoxyphenol) and intermediate **11** with an energy barrier of 247.3 kJ/mol, through which the H atom of the hydroxyl group at C_α_ position transfers to the oxygen atom of the ether bond. Intermediate **11** then undergoes transition state **ts12** to form product **3** (3-hydroxy-1-phenylpropan-1-one) with an energy barrier of 292.8 kJ/mol.

In concerted mechanism 6, the H atom of the hydroxyl group at C_α_ position of α-OH-β-CH_2_OH-*o*-CH_3_O-PPE transfers to C_β_ position and the C_α_–C_β_ bond breaks to form intermediate **12** and product **13** (benzaldehyde) with an energy barrier of 363.1 kJ/mol. Intermediate **12** goes through a four membered ring transition state **ts14** to form product **2** (2-methoxyphenol) and intermediate **14** with an energy barrier of 287.8 kJ/mol. Intermediate **14** will undergo isomerization through transition state **ts15** to form product **15** (acetaldehyde) with an energy barrier of 224.5 kJ/mol.

Mechanisms 7 and 8 are closely related to the hydroxymethyl group at C_β_ position. In concerted mechanism 7, the H atom of the hydroxyl group at C_γ_ position of α-OH-β-CH_2_OH-*o*-CH_3_O-PPE transfers to the oxygen atom of the ether bond through transition state **ts16** to generate product **2** (2-methoxyphenol) and intermediate **16**, overcoming an energy barrier of 267.4 kJ/mol. The hydroxyl group at C_α_ position and the H atom at C_β_ position of intermediate **16** undergo dehydration reaction with an energy barrier of 228.0 kJ/mol, forming product **17** (3-phenylacrylaldehyde).

In concerted mechanism 8, α-OH-β-CH_2_OH-*o*-CH_3_O-PPE goes through transition state **ts18** to generate product **2** (2-methoxyphenol) and intermediate **18** with an energy barrier of 250.4 kJ/mol, through which the H atom at C_γ_ position transfers to the oxygen atom of the ether bond. The enol structure of intermediate **18** is easy to undergo isomerization through transition state **ts19** to form intermediate **16** with an energy barrier of 242.9 kJ/mol. Then intermediate **16** will transform into product **17** (3-phenylacrylaldehyde) following the reaction pathway in concerted mechanism 7. In addition to the isomerization reaction, intermediate **18** can also undergo dehydration reaction through a six-membered ring transition state **ts20** to form product **17** (3-phenylacrylaldehyde) with an energy barrier of 87.6 kJ/mol, which is much lower than the energy barrier of **ts19**. Therefore, it is much easier for intermediate **18** to go through the dehydration reaction to form product **17**.

Concerted mechanism 9 is related to both the hydroxyl group at C_α_ position and the hydroxymethyl group at C_β_ position. α-OH-β-CH_2_OH-*o*-CH_3_O-PPE undergoes a six membered ring transition state **ts21** to directly form product **10** (1-methoxy-2-(styryloxy)benzene) with an energy barrier of 267.5 kJ/mol, losing a molecule of water and formaldehyde.

According to the above results, the sequence of energy barriers of the above nine concerted mechanisms is as follows: mechanism 2 < mechanism 3 < mechanism 5 < mechanism 8 < mechanism 1 < mechanism 7 < mechanism 9 < mechanism 4 < mechanism 6. The primary aromatic products are **2** (2-methoxyphenol), **3** (3-hydroxy-1-phenylpropan-1-one), **8** (phenol), **17** (3-phenylacrylaldehyde), **10** (1-methoxy-2-(styryloxy)benzene) and **13** (benzaldehyde), as well as some low molecular compounds (formaldehyde, water and acetaldehyde). As shown in Figure 3, these products are formed via different mechanisms. Products **2** and **3** can be formed from mechanisms 1, 2 and 5, among which the energy barrier of mechanism 2 is the lowest. Mechanism 8 is superior to mechanism 7 to form product **17**, and mechanism 9 is superior to mechanism 4 to form product **10**. In regard to products **8** and **13**, their formation pathways are attributed to mechanisms 3 and 6, respectively.

#### 2.2.2. Homolytic Mechanisms of Model Compound α-OH-β-CH_2_OH-*o*-CH_3_O-PPE

**Figure 4:**
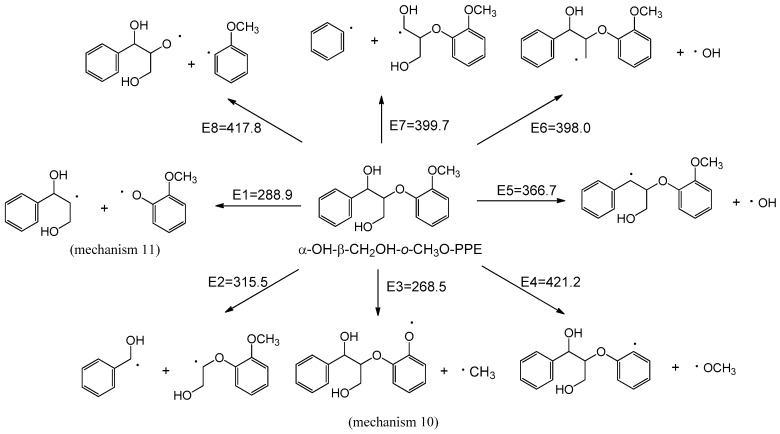
Bond dissociation energies of the major bonds in lignin model compound α-OH-β-CH_2_OH-*o*-CH_3_O-PPE (unit: kJ/mol).

The possible homolytic cleavage reactions of the major bonds and their corresponding BDEs are illustrated in Figure 4. Compared with other homolytic cleavage reactions, the O–CH_3_ bond homolytic cleavage (mechanism 10) has the lowest BDE, followed by the C_β_–O bond homolytic cleavage (mechanism 11), indicating that mechanisms 10 and 11 are the two favorable homolytic mechanisms of lignin model compound α-OH-β-CH_2_OH-*o*-CH_3_O-PPE. Other homolytic cleavage reactions can hardly take place due to the high BDEs. Such results also reflect that the proposed pyrolysis model in Figure 1 is reasonable. Except for concerted mechanisms 4 and 6, the activation energies of other concerted mechanisms are lower than the BDEs of homolytic mechanisms during the primary pyrolysis process. Hence, it can be concluded that concerted mechanisms should be mainly responsible for the pyrolysis process, while homolytic mechanisms are less important than concerted mechanisms. The subsequent pyrolysis pathways based on mechanisms 10 and 11 are discussed below.

**Figure 5:**
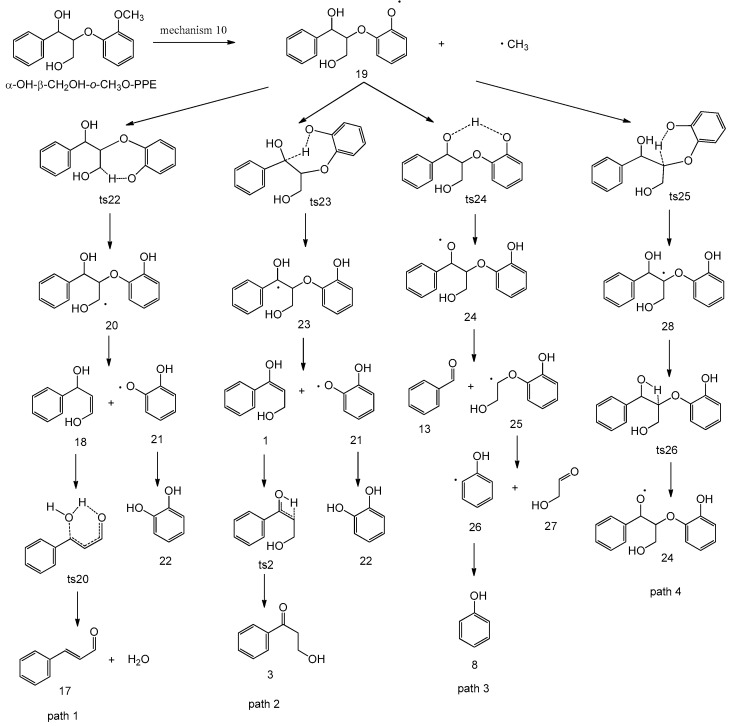
Possible reaction pathways based on O–CH_3_ bond homolytic cleavage of α-OH-β-CH_2_OH-*o*-CH_3_O-PPE.

**Figure 6:**
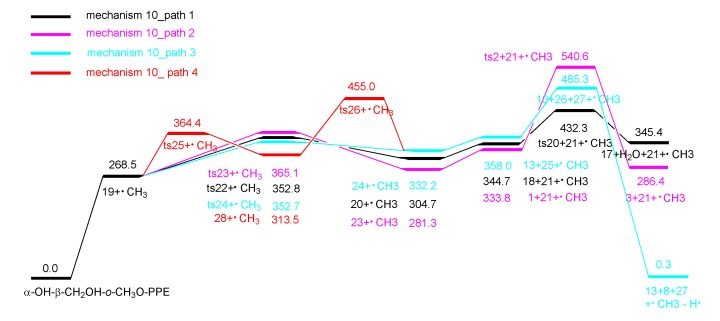
Potential energy profile along reaction pathways for the homolytic cleavage of O–CH_3_ bond.

In homolytic mechanism 10, α-OH-β-CH_2_OH-*o*-CH_3_O-PPE undergoes O–CH_3_ bond homolytic cleavage to form radical **19** and methyl radical with an energy barrier of 268.5 kJ/mol. As shown in Figure 5, radical **19** has four possible subsequent reaction pathways. The potential energy profile along reaction pathways for the homolytic cleavage of O–CH_3_ bond is shown in Figure 6.

In path 1, the H atom at C_γ_ position of radical **19** transfers to phenoxy radical through a seven-membered ring transition state **ts22** to form radical **20** with an energy barrier of 84.3 kJ/mol. Radical **20** undergoes further C_β_–O bond homolytic cleavage to form intermediate **18** and radical **21** with an energy barrier of 40.0 kJ/mol. Radical **21** will couple with free H radical to form hydrogenated product **22** (catechol). Intermediate **18** will transform into product **17** (3-phenylacrylaldehyde) following the reaction pathway in concerted mechanism 8.

In path 2, the H atom at C_α_ position of radical **19** transfers to phenoxy radical through a seven-membered ring transition state **ts23** to form radical **23** with an energy barrier of 96.6 kJ/mol. Radical **23** further undergoes C_β_–O bond homolytic cleavage to generate intermediate **1** and radical **21** with an energy barrier of 52.5 kJ/mol. Intermediate **1** will transform into product **3** (3-hydroxy-1-phenylpropan-1-one) following the reaction pathway in concerted mechanism 1.

In path 3, the H atom of the hydroxyl group at C_α_ position of radical **19** transfers to phenoxy radical through an eight-membered ring transition state **ts24** to form radical **24** with an energy barrier of 84.2 kJ/mol. Then radical **24** undergoes C_α_–C_β_ bond homolytic cleavage to form product **13** (benzaldehyde) and radical **25** with an energy barrier of 25.8 kJ/mol. Radical **25** continues to undergo C_aromatic_–O bond homolytic cleavage to form radical **26** and product **27** (hydroxyacetaldehyde) with an energy barrier of 127.3 kJ/mol. Radical **26** will combine with free H radical to form hydrogenated product **8** (phenol).

In path 4, radical **19** undergoes intramolecular hydrogen transfer reactions through two consecutive transition states **ts25** and **ts26** to form radical **24** with energy barriers of 95.9 kJ/mol and 141.5 kJ/mol, respectively. Radical **24** will follow path 3 to undergo subsequent reactions. Comparing the energy barriers to form radical **24** in paths 3 and 4, path 3 is superior to path 4 due to its lower energy barrier.

The above four subsequent pyrolysis pathways based on homolytic mechanism 10 are competitive with each other. As shown in Figure 6, the energy barriers of **ts22** and **ts24** are close and lower than those of **ts23** and **ts25**. Hence, the major pyrolytic products based on mechanism 10 include **17** (3-phenylacrylaldehyde), **22** (catechol), **13** (benzaldehyde), **8** (phenol) and **27** (hydroxyacetaldehyde). Products **17** and **13** can also be formed through concerted mechanisms 8 and 6, respectively. In comparison of concerted mechanism 8 and homolytic mechanism 10, product **17** is easily formed from concerted mechanism 8, while product **13** is easily formed from homolytic mechanism 10.

**Figure 7:**
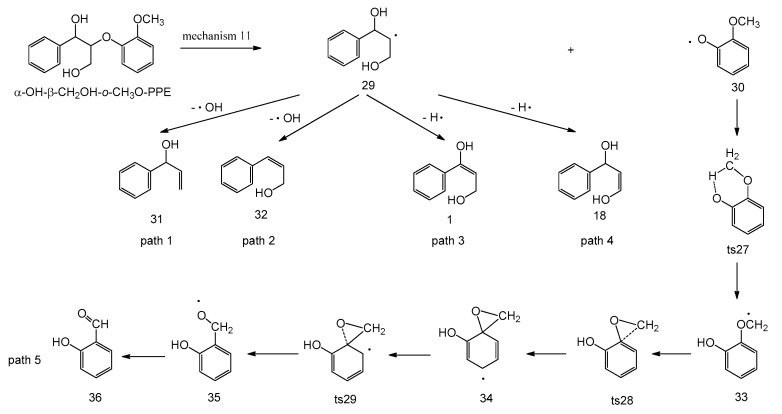
Possible reaction pathways based on C_β_–O bond homolytic cleavage of α-OH-β-CH_2_OH-*o*-CH_3_O-PPE.

**Figure 8:**
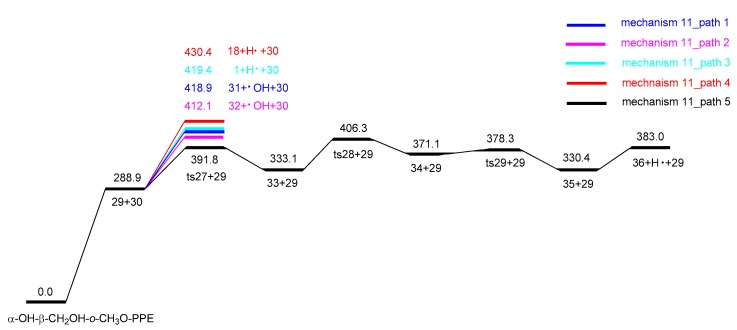
Potential energy profile along reaction pathways for the homolytic cleavage of C_β_–O bond (unit: kJ/mol).

In homolytic mechanism 11, α-OH-β-CH_2_OH-*o*-CH_3_O-PPE undergoes C_β_–O bond homolytic cleavage to form radicals **29** and **30** with an energy barrier of 288.9 kJ/mol. The subsequent pyrolysis pathways of radicals **29** and **30** are shown in Figure 7, and the corresponding potential energy profile is depicted in Figure 8. Radical **29** has four possible following pathways. It loses the hydroxyl groups at C_γ_ and C_α_ positions to form products **31** (1-phenylprop-2-en-1-ol) and **32** (3-phenylprop-2-en-1-ol) with energy barriers of 130.0 kJ/mol and 123.2 kJ/mol, respectively. Or it undergoes dehydrogenation reactions at C_α_ and C_γ_ positions to form intermediates **1** and **18** with energy barriers of 130.5 kJ/mol and 141.5 kJ/mol, respectively. They will follow the reaction pathways in concerted mechanisms 1 and 8 to form products **3** (3-hydroxy-1-phenylpropan-1-one) and **17** (3-phenylacrylaldehyde). As shown in Figure 8, the above four pathways of radical **29** are competitive with each other, among which path 2 has the lowest energy barrier to form product **32** (3-phenylprop-2-en-1-ol).

Radical **30** can undergo a series of reactions to form product **36** (2-hydroxybenzaldehyde). It firstly undergoes intramolecular hydrogen transfer reaction through a six-membered ring transition state **ts27** to form radical **33** with an energy barrier of 102.9 kJ/mol. Radical **33** then goes through C–O shift reaction via two consecutive transition states **ts28** and **ts29** to form radical **35** with energy barriers of 73.2 kJ/mol and 7.2 kJ/mol, respectively. Radical **35** finally undergoes dehydrogenation reaction to form product **36** (2-hydroxybenzaldehyde) with an energy barrier of 52.6 kJ/mol. Hence, the major pyrolytic products based on homolytic mechanism 11 (paths 2 and 5) are **32** (3-phenylprop-2-en-1-ol) and **36** (2-hydroxybenzaldehyde).

#### 2.2.3. Summary

Based on the energy barriers of the above 11 pyrolysis mechanisms of α-OH-β-CH_2_OH-*o*-CH_3_O-PPE, their competitiveness will follow this order, i.e., mechanism 2 > mechanism 3 > mechanism 5 > mechanism 8 > mechanism 1 > mechanism 7 > mechanism 9 > mechanism 10 > mechanism 11 > mechanism 4 > mechanism 6. Among the unimolecular decomposition pathways of α-OH-β-CH_2_OH-*o*-CH_3_O-PPE, most of the concerted mechanisms are superior to homolytic mechanisms except for mechanism 6. The major pyrolytic products based on concerted mechanisms include **2** (2-methoxyphenol), **3** (3-hydroxy-1-phenylpropan-1-one), **8** (phenol), **10** (1-methoxy-2-(styryloxy)benzene) and **17** (3-phenylacrylaldehyde). While the pyrolytic products based on O–CH_3_ and C_β_–O bond homolytic mechanisms include **13** (benzaldehyde), **22** (catechol), **32** (3-phenylprop-2-en-1-ol) and **36** (2-hydroxybenzaldehyde). Product **2** (2-methoxyphenol) is the most abundant product generated from most of the concerted mechanisms with relatively low energy barriers. In addition, radical **30** formed from C_β_–O bond homolytic mechanism can also combine with free H radical and undergo H-abstraction to form product **2** (2-methoxyphenol) [20,23,30]. It agrees with the results obtained by He et al. [22] and Jiang et al. [31]. They conducted pyrolysis experiments on a β-*O*-4 type lignin dimer model compound (guaiacylglycerol-β-guaiacyl ether) which has same functional groups on the alkyl side chain and aromatic ring near the ether bond. They found that 2-methoxyphenol was the major product while the other products were in low yields. These experimental results firmly confirm that the proposed pyrolysis model in Figure 1 is reasonable and reliable.

The integrated pyrolysis mechanisms of the other four β-*O*-4 type lignin model compounds PPE, α-OH-PPE, β-CH_2_OH-PPE and *o*-CH_3_O-PPE are depicted in the Supplementary Materials (Figures S1–S4) based on the proposed pyrolysis model in Figure 1. Pyrolysis of PPE may follow mechanisms 1, 2 and 11 to decompose, and their competitiveness is in the order of mechanism 2 > mechanism 1 > mechanism 11. α-OH-PPE may undergo pyrolysis reactions through mechanisms 1, 2, 4, 5, 6 and 11, and their competitiveness follows the order of mechanism 2 > mechanism 1 > mechanism 4 > mechanism 11 > mechanism 5 > mechanism 6. β-CH_2_OH-PPE may follow five pyrolysis mechanisms to decompose, whose competitiveness order is mechanism 2 > mechanism 1 > mechanism 7 > mechanism 8 > mechanism 11. *o*-CH_3_O-PPE also has five possible pyrolysis mehanisms with the competitiveness order of mechanism 2 > mechanism 3 > mechanism 1 > mechanism 10 > mechanism 11. It is important to note that the major pyrolytic products of the four model compounds obtained through DFT calculations shown in the Supplementary Materials agree well with the experimental results [18,19,29,31], which futher confirms the validity of the pyrolysis model. Based on the results, a similar conclusion can be drawn that most of the concerted mechanisms are prior to homolytic mechanisms, except for mechanism 6. The above results clearly illustrate that based on the pyrolysis model proposed in Figure 1, the pyrolysis mechanisms for different β-*O*-4 type lignin dimer model compounds basically follow certain specific rules, but different functional groups will affect the pyrolytic product distribution. Hence, the pyrolysis model in Figure 1 can be used to predict the pyrolytic pathways and products.

## 3. Discussion

According to the above analyses, concerted mechanisms and homolytic mechanisms are coexisting in the pyrolysis process of β-*O*-4 type lignin dimer model compounds. Moreover, concerted mechanisms are generally prior to homolytic mechanisms, indicating concerted mechanisms are dominant for the pyrolysis process. Mechanisms 1, 2 and 11 are fundamental for all β-*O*-4 type lignin dimer model compounds and their competitiveness follows the order of mechanism 2 > mechanism 1 > mechanism 11, which is irrelevant to the hydroxyl, hydroxymethyl and methoxyl groups. In consideration of the effects of functional groups, the competitiveness of the proposed 11 pyrolysis mechanisms for different β-*O*-4 type lignin dimers can be concluded in the order of mechanism 2 > mechanism 3 > mechanism 1 > mechanism 7 > mechanism 9 > mechanism 10 > mechanism 11 > mechanism 6, wherein mechanisms 4, 5 and 8 can hardly be ranked because their competitiveness is dramatically affected by the functional groups on the aromatic ring and alkyl side chain. Among these mechanisms, mechanism 3 is quite competitive with mechanism 2 (retro-ene fragmentation) which is considered as the dominant pyrolysis mechanism of substituted β-*O*-4 model compounds [21,23]. Mechanism 10 (O–CH_3_ bond homolytic cleavage) will supersede mechanism 11 (C_β_–O bond homolytic cleavage) to be the optimal homolytic mechanism. Mechanism 6 can hardly take place due to its high energy barrier.

The application of the proposed pyrolysis model ultimately offers a theoretical basis to predict the pyrolytic pathways and the formed products. PPE is the simplest β-*O*-4 type lignin dimer model compound [32], and the major pyrolytic products are styrene and phenol, which has been confirmed in this study as well as previous studies [29,33,34]. In the presence of functional groups on the alkyl side chain and aromatic ring, the pyrolytic product distribution will be changed, but have regularity. Pyrolysis of the methoxyl-substituted PPEs mainly proceeds through concerted mechanisms (mechanisms 1, 2, 5, 7 and 8) to produce 2-methoxyphenol (or 2,6-dimethoxyphenol) as one of the dominant pyrolytic products. Furthermore, another dominant product 2-hydroxybenzaldehyde (or 2-hydroxy-3-methoxybenzaldehyde) can be formed from a key intermediate methoxyphenoxy (or 2,6-dimethoxyphenoxy) radical through a series of reaction steps [2,29] shown in Figure 7 (mechanism 11). The α-hydroxyl-substituted PPEs may undergo dehydration reactions to form the compounds containing an unsaturated C=C double bond through mechanisms 4. These compounds are easy to transform into macromolecular compounds and coke through polymerization [35], which may be one of the possible reasons for high char yield during lignin pyrolysis process. In addition, the major pyrolytic products from pyrolysis of α-hydroxyl-substituted PPEs have a C=O feature structure at C_α_ position. Similarly, the major pyrolytic products formed from pyrolysis of β-hydroxymethyl-substituted PPEs also have a C=O feature structure at C_γ_ position. In the following studies, the pyrolysis models for other lignin structures containing oxidized substituents and α-*O*-4, 4-*O*-5, β-1, β-5 or 5-5 linkages will be investigated to comprehensively understand the lignin pyrolysis process, which can provide theoretical guidance for developing efficient selective pyrolysis techniques.

## 4. Calculation Methods

All calculations were conducted in Gaussian 09 suite of programs [36]. The geometries of reactants, intermediates, transition states and products were optimized by using hybrid density functional M06-2X and the 6−31+G(d,p) basis set, followed by frequency calculations. To simulate the real pyrolysis condition, the thermodynamic parameters at typical pyrolysis temperature and pressure (873 K and 101 kPa) were obtained by frequency analyses. The transition states were located by TS method in which only the initial guesses of the transition states were used without using the corresponding initial guesses of reactants and products. Furthermore, intrinsic reaction coordinate (IRC) calculations were conducted to confirm the corresponding reactants, transition states and products on the same potential energy surface (PES). The reactants, intermediates and products had no imaginary frequency, while the transition states had only one imaginary frequency. Activation energies for concerted reactions were estimated with the energy differences between transition states and reactants. While for homolytic cleavage reactions, transition states were very difficult to find because there were no saddle points on the PES, the BDE was generally regarded as approximation to activation energy for comparison [13,34,37].

## 5. Conclusions

A pyrolysis model containing 11 initial pyrolysis mechanisms (mechanisms 1–11) for β-*O*-4 type lignin dimers is proposed which comprehensively considers the effects of functional groups (hydroxyl, hydroxymethyl and methoxyl) on the alkyl side chain and aromatic ring. Based on the pyrolysis model, the integrated pyrolysis mechanisms of five specific β-O-4 type lignin dimer model compounds are investigated by DFT calculations. The results indicate that among the unimolecular decomposition pathways, concerted mechanisms and homolytic mechanisms are coexisting, while concerted mechanisms are predominant and mainly responsible for the pyrolysis process. Mechanisms 1, 2 and 11 are three basic mechanisms of all β-*O*-4 type lignin dimers and their competitiveness order remains invariable (mechanism 2 > mechanism 1 > mechanism 11) regardless of functional groups. The competitiveness of the proposed 11 pyrolysis mechanisms follows the order of mechanism 2 > mechanism 3 > mechanism 1 > mechanism 7 > mechanism 9 > mechanism 10 > mechanism 11 > mechanism 6, wherein mechanisms 4, 5 and 8 can hardly be ranked in view of the effects of functional groups. Combining previous experimental studies, it can be concluded that the proposed pyrolysis model is reasonable and reliable.

## Supplementary Materials

Supplementary materials can be found at www.mdpi.com/1422-0067/18/11/2364/s1.
