# Supplementary material for: A Comprehensive Study on Pyrolysis Mechanism of Substituted β-O-4 Type Lignin Dimers
Source: Int J Mol Sci. 2017 Nov 9;18(11):2364. doi: 10.3390/ijms18112364 (PMC5713333; doi:10.3390/ijms18112364)
Supplement: Supplementary file 1 [file ijms-18-02364-s001.pdf]

Supplementary Materials

A comprehensive study on pyrolysis mechanism of substituted β-O-4 type lignin dimers

Xiaoyan Jiang, Qiang Lu*, Bin Hu, Ji Liu, Changqing Dong and Yongping Yang

National Engineering Laboratory for Biomass Power Generation Equipment, North China Electric Power University, Beijing 102206, China; jiangxiaoyan@ncepu.edu.cn (X.J.); hubin@ncepu.edu.cn (B.H.); liujipower@ncepu.edu.cn (J.L.); dongcq@ncepu.edu.cn (C.D.); yyp@ncepu.edu.cn (Y.Y.)

* Correspondence: qlu@ncepu.edu.cn; Tel.: +86-10-6177-2030

Based on the pyrolysis model proposed in Figure 1, the integrated pyrolysis mechanisms of other four β-O-4 type lignin dimer model compounds (phenethyl phenyl ether (PPE), 1-methoxy-2-phenethoxybenzene (*o*-CH_3O-PPE), 2-phenoxy-1-phenylethanol (*α*-OH-PPE), 2-phenoxy-3-phenylpropan-1-ol (β-CH_2OH-PPE)) are investigated, and their pyrolysis pathways and products are shown in Figures S1, S2, S3 and S4, respectively. In addition, the calculation results correspond well with previous experimental studies [18,19,29,31].

[18] Zhang, J.J.; Jiang, X.Y.; Ye, X.N; Chen, L.; Lu, Q.; Wang, X.H; Dong, C.Q. Pyrolysis mechanism of a β-O-4 type lignin dimer model compound. *J. Therm. Anal. Calorim.* **2016,** *123*, 501-510, DOI: 10.1007/s10973-015-4944-y.

[19] Chen, L.; Ye, X.; Luo, F.; Shao, J.; Lu, Q.; Fang, Y.; Wang, X.; Chen, H. Pyrolysis mechanism of β-O-4 type lignin model dimer. *J. Anal. Appl. Pyrol.* **2015,** *115*, 103-111, DOI: 10.1016/j.jaap.2015.07.009.

[29] Britt, P.F.; Buchanan, A.C.; Cooney, M.J.; Martineau, D. R. Flash vacuum pyrolysis of methoxy-substituted lignin model compounds. *J. Org. Chem.* **2000,** *65*, 1376-1389, DOI: 10.1021/jo991479k.

[31] Jiang, W.; Wu, S.; Lucia, L.A.; Chu, J. A comparison of the pyrolysis behavior of selected β-O-4 type lignin model compounds. *J. Anal. Appl. Pyrol.* **2017,** *125*, 185-192, DOI: 10.1016/j.jaap.2017.04.003.

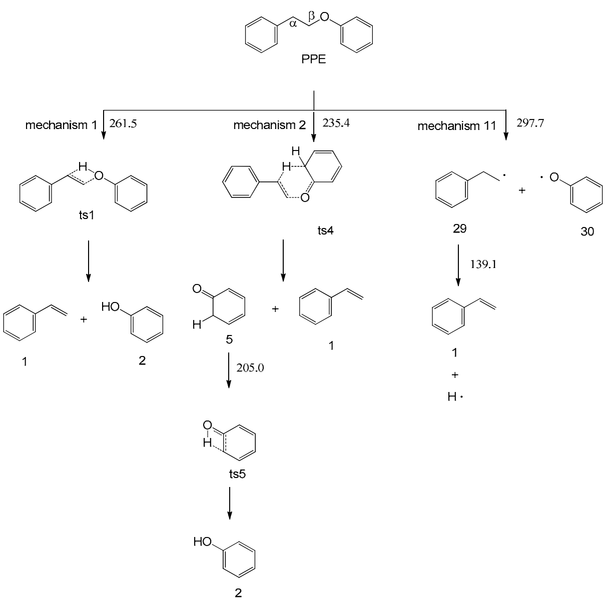

Figure S1. The integrated pyrolysis mechanism of model compound PPE (unit: kJ/mol).

Figure S1 shows the integrated pyrolysis mechanism of model compound PPE and energy barriers for the reaction steps in pyrolysis pathways. According to Figure S1, PPE mainly undergoes mechanisms 1, 2 and 11 to form the major pyrolytic products of styrene and phenol, which agrees well with the experimental results obtained by Britt et al. [29].

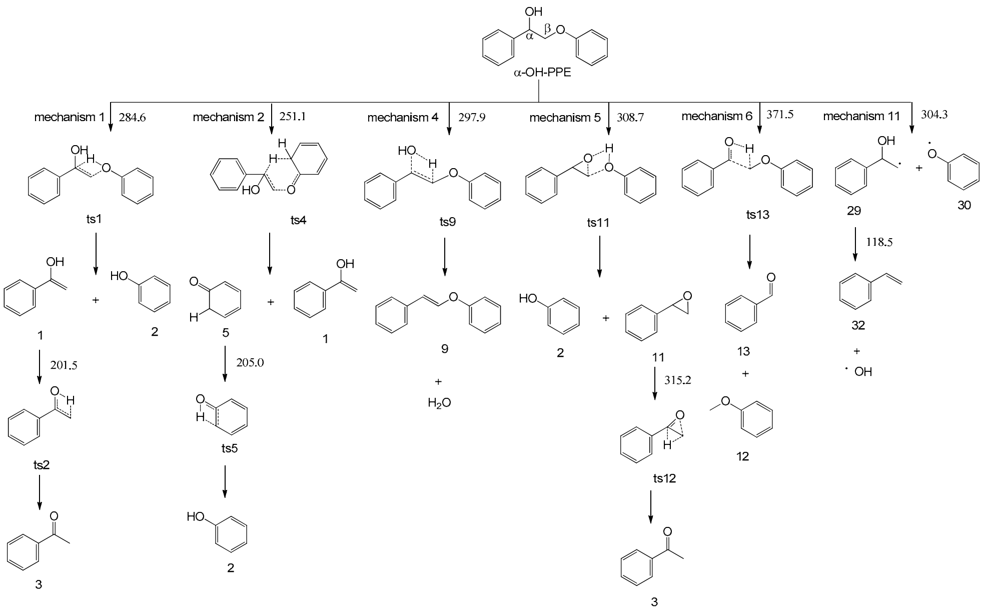

Figure S2. The integrated pyrolysis mechanism of model compound α-OH-PPE (unit: kJ/mol).

Figure S2 shows the integrated pyrolysis mechanism of model compound α-OH-PPE and energy barriers for the reaction steps in pyrolysis pathways. According to Figure S2, α-OH-PPE mainly undergoes mechanisms 1, 2, 4, 5 and 11 to form the major pyrolytic products of phenol, acetophenone, styrene and 2-phenoxyvinylbenzene. Mechanism 6 is non-competitive due to its high energy barrier. The theoretical calculation results correspond with the experimental results obtained by Jiang et al. [31] and Chen et al. [19].

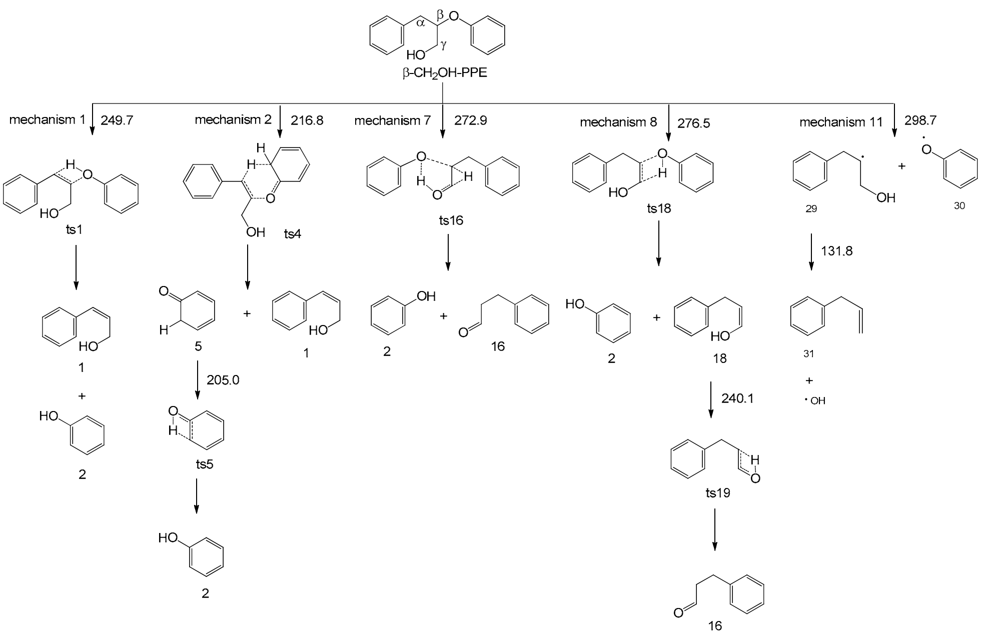

Figure S3. The integrated pyrolysis mechanism of model compound β-CH_2OH-PPE (unit: kJ/mol).

Figure S3 shows the integrated pyrolysis mechanism of model compound β-CH_2OH-PPE and energy barriers for the reaction steps in pyrolysis pathways. According to Figure S3, β-CH_2OH-PPE mainly undergoes mechanisms 1, 2, 7, 8 and 11 to form the major pyrolytic products of phenol, 3-phenylprop-2-en-1-ol, 3-phenylpropanal and allylbenzene.

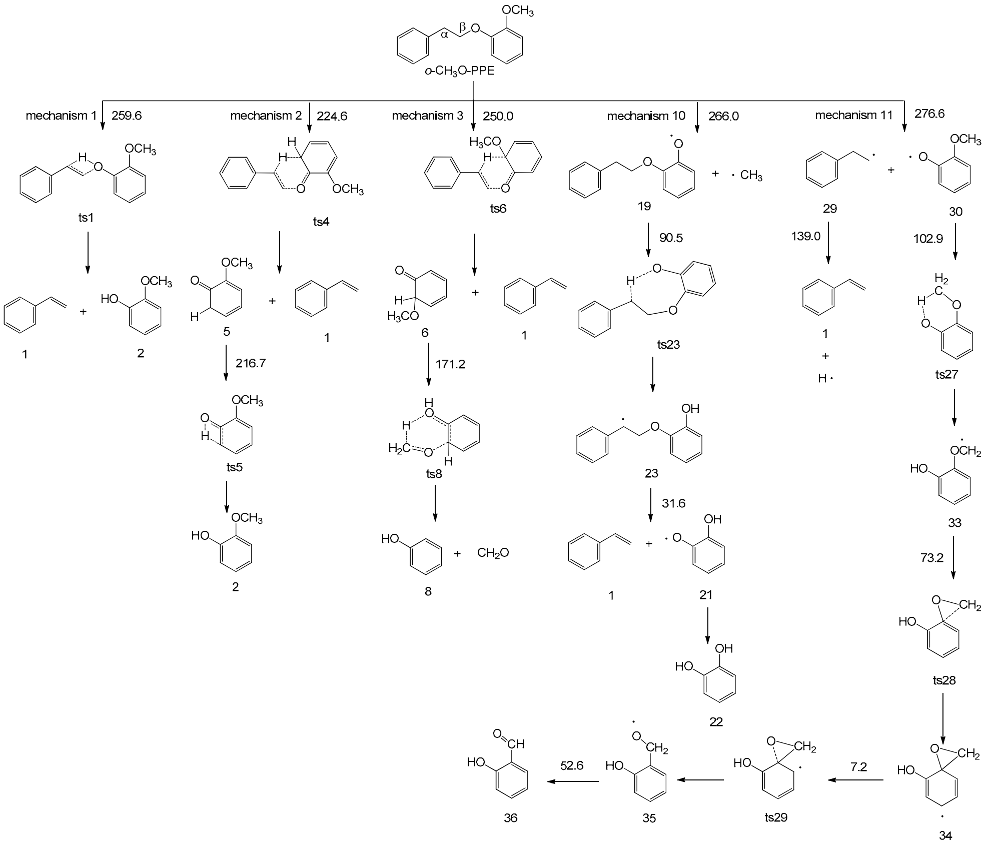

Figure S4. The integrated pyrolysis mechanism of model compound *o*-CH_3O-PPE (unit: kJ/mol).

Figure S4 shows the integrated pyrolysis mechanism of model compound *o*-CH_3O-PPE and energy barriers for the reaction steps in pyrolysis pathways. According to Figure S4, *o*-CH_3O-PPE mainly undergoes mechanisms 1, 2, 3, 10 and 11 to form the major pyrolytic products of styrene, 2-methoxyphenol, phenol, catechol and 2-hydroxybenzaldehyde, which agrees with the literature results [18,29].
